# Influence of high-irradiance light curing on the marginal integrity of composite restorations in primary teeth

**DOI:** 10.1186/s12903-023-03291-6

**Published:** 2023-08-13

**Authors:** Janina Frank, Tobias T. Tauböck, Marcus Zimmermann, Thomas Attin, Blend Hamza

**Affiliations:** 1 Clinic of Conservative and Preventive Dentistry, Center of Dental Medicine, University of Zurich, Plattenstrasse 11, Zurich, 8032 Switzerland; 2 Clinic of Orthodontics and Pediatric Dentistry, Center of Dental Medicine, University of Zurich, Plattenstrasse 11, Zürich, 8032 Switzerland

**Keywords:** Rapid high-irradiance light-curing, Bulk-fill composite, Primary molars, Marginal integrity, Scanning electron microscopy

## Abstract

**Background:**

Reducing the necessary time to restore primary teeth improves the cooperation of paediatric patients. This study aimed to investigate the marginal integrity of restorations prepared with a bulk-fill resin-based composite (RBC) containing additional fragmentation chain transfer (AFCT) compared to a conventional RBC when light cured with a rapid high-irradiance (3 s) and a regular (10 s) curing mode.

**Methods:**

Forty class-II cavities were prepared in 40 primary molars. The molars were randomly divided into four groups based on the applied light-curing modes (regular: 10 s @ 1200 mW/cm^2^ or high-irradiance: 3 s @ 3000 mW/cm^2^) and the used restorative material (AFCT-containing bulk-fill RBC “Power Fill” or AFCT-free conventional RBC “Prime”). After thermo-mechanical loading, the marginal integrity was analysed using scanning electron microscopy. A beta regression model and pairwise comparisons were used to statistically analyse the data.

**Results:**

The mean marginal integrity (% ± SD) of the restorations for each group was as follows: Power Fill (10 s: 79.7 ± 15.6) (3 s: 77.6 ± 11.3), Prime (10 s: 69.7 ± 11.1) (3 s: 75.0 ± 9.7). The difference between the RBCs for the same light-curing mode was statistically significant (*p* ≤ 0.05). The difference between the light-curing modes for the same RBC was not statistically significant (*p* ˃ 0.5).

**Conclusions:**

AFCT-containing bulk-fill RBC “Power Fill” achieves similar marginal integrity when light-cured with either high-irradiance or regular light-curing modes. “Power Fill” achieves better marginal integrity than the conventional RBC “Prime” regardless of the applied light-curing mode.

## Background

Resin-based composites have become a solid restorative material option in both permanent and primary teeth [1–3]. Some reports considered the classical incremental layering technique (i.e., the consecutive application of 2-mm-thick composite layers into the tooth cavity) as complicated and connected it with possible air entrapment between the layers and long treatment duration [3]. As an attempt to simplify and shorten the restoration procedure, bulk-fill composites, which can be applied in 4–5 mm layers, were introduced and proven to be a valid alternative to conventional composites, both in vitro and in vivo [4–6]. This comparable good performance of bulk-fill composites was attributed to improved depth of cure and shrinkage stress [7, 8]. Yet another attempt was made to shorten the treatment duration by introducing bulk-fill composites that require much less photo-polymerisation time, namely only 3 s instead of other polymerisation times that usually begin at 10 s [9].

Two major developments led to the possibility of such short polymerisation time: the introduction of high-power light emitting diode (LED) polymerisation units that can produce high radiant exitance (e.g., 3000 mW/cm^2^), and the incorporation of an β-allyl sulfone addition fragmentation chain transfer (AFCT) reagent in the matrix of the bulk-fill composite [9, 10]. AFCT reagent is supposed to regulate the radical polymerisation reaction of the composite matrix [11]. In other words, when an AFCT-free composite is photo-polymerised, composite monomers, usually methacrylate groups, will rapidly bond to free radicals. This reaction chain progresses rapidly with more monomers being incorporated into the growing polymer network until the concentration of available monomers decreases and the radical chain cannot continue to grow through the gel-becoming composite matrix [12]. This results in unreacted monomers being trapped within the polymer network and hence to irregular, long-chained and brittle network [11]. On the other hand, the presence of AFCT reagents prevents the formation of the mentioned long chains and promotes a step-like growth of the polymer chain enhancing the homogeneity of the polymer network and its thermal and mechanical properties [11, 13].

It has been reported that children show more behaviour difficulties with an increase of the treatment duration [14]. Therefore, the aforementioned shortening of treatment duration and simplifying the restoration procedure could bring important benefits in paediatric dentistry. However, the performance of composite restorations photoactivated with high-irradiance light-curing modes has not yet been investigated on primary molars. This in-vitro study was therefore carried out to investigate and compare the marginal integrity of an AFCT-containing bulk-fill RBC (Power Fill “high-viscous”, Ivoclar Vivadent, Schaan, Liechtenstein) and a conventional RBC (Prime “high-viscous”, Ivoclar Vivadent) when light-cured using regular and high-irradiance light-curing modes in primary molars. The first null-hypothesis was that the light-curing mode (high-irradiance for 3 s compared to regular for 10 s) would have no effect on the marginal integrity. The second null-hypothesis was that the tested RBCs (AFCT-containing bulk-fill RBC compared to conventional RBC) would have no effect on the marginal integrity regardless of the used light-curing mode.

## Materials and methods

Forty first and second human primary molars from the upper and lower jaw with one sound proximal surface were included in this study. The molars were extracted due to apical periodontitis or orthodontic reasons and stored in 0.5% chloramine-T solution at 4 °C for no longer than two months. Children and parents gave their written consent for the use of the molars for research purposes. After extraction, all molars were irreversibly anonymised. Therefore, this study was carried out in agreement with the Federal Act on Research involving Human Beings (Human Research Act; article 2, paragraph 2) and the authorisation from the ethics committee was waived (Zurich cantonal ethics commission, BASEC-2022-00962). Roots of the extracted molars were embedded in acrylic resin (Paladur, Heraeus Kulzer, Hanau, Germany) 3 mm below the cemento-enamel junction and mounted on custom-made holders. One standardised mesial or distal proximal cavity (3 mm in width, 1.5 mm in axial depth, with cervical margins 1 mm below the cemento-enamel junction) was prepared in each primary molar. Proximal cavities were prepared with 80-µm cylindrical burs (Universal Prep Set, Intensiv, Grancia, Switzerland) rotating at 40,000 rpm in a high-speed contra-angle handpiece (Sirius, Micro-Mega, Besancon Cedex, France). The bur was exchanged after preparing four cavities. The molars were then randomised into four groups (n = 10, computer-generated randomisation table, Microsoft Excel) based on the RBC they would be restored with and the light-curing mode they would be subjected to. The sample size was determined based on recent similar research [5, 6].

**Fig. 1:**
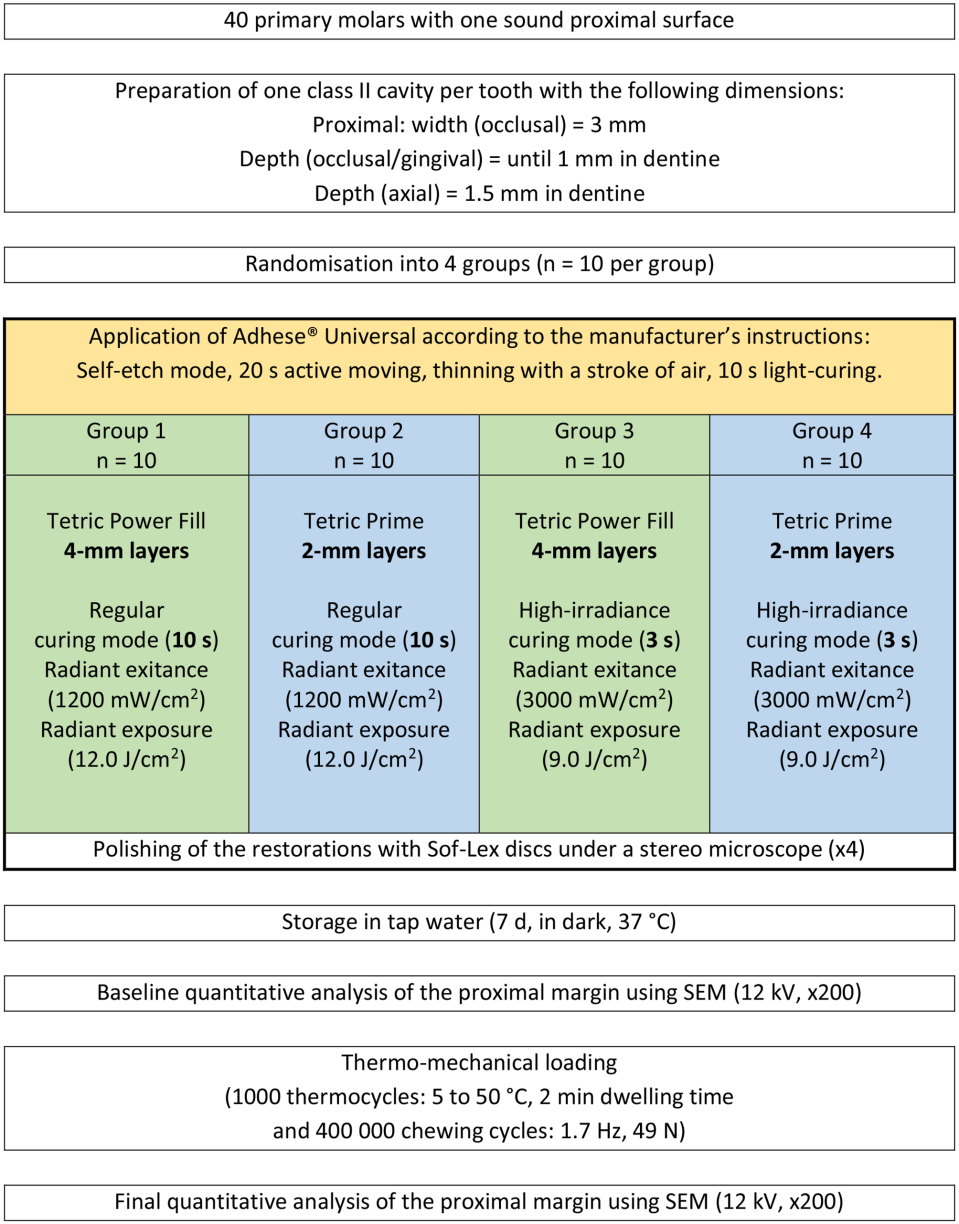
Study design

The molars were mounted and restored in a custom-made adjacent-tooth simulator with two plastic molars on each side on a laboratory desk. A stainless-steel matrix band (Omni-Matrix sectional, extended, Ultradent Products, South Jordan, UT, USA) was inserted and fixed with a wooden wedge placed 1 mm below the gingival margin of the cavity. A universal adhesive (Adhese Universal, Ivoclar Vivadent) was scrubbed for 20 s on all cavity walls in self-etch mode, thinned with a gentle blow of air in order to evaporate the solvent, and then light-cured for 10 s at 1200 mW/cm^2^ (Bluephase PowerCure, Ivoclar Vivadent). The cavities were restored based on their experimental group as shown in Fig. 1. Two RBCs were used to restore the primary molars: Power Fill, an AFCT-containing high-viscous bulk-fill composite that was applied in 4-mm thick layers, and Prime, a conventional composite that was applied in 2-mm thick layers. In groups 1 and 2, each layer of both materials was subjected to regular light-curing (radiant exitance = 1200 mW/cm^2^, curing time = 10 s). In groups 3 and 4, each layer of both materials was subjected to high-irradiance light-curing (radiant exitance = 3000 mW/cm^2^, curing time = 3 s). The composition of the used RBCs is shown in Table 1. The tip of the curing unit (diameter = 9 mm) was always placed as near as possible to the surface to be light cured (in direct contact with the roof of the cavity when light-curing the first layer/layers and at a distance of 1 mm from the RBC surface when light-curing the last layer). The radiant exitance values were measured and periodically controlled using a calibrated and NIST-referenced UV–Vis spectrophotometer system (MARC; BlueLight Analytics, Halifax, Canada). Under a stereo microscope (× 4), all restorations were polished using Sof-Lex discs with decreasing grit-sizes (Sof-Lex Pop-on, 3 M ESPE, St. Paul, MN, USA) under constant water cooling. Sof-Lex discs were replaced after polishing four restorations. The filled molars were immersed in tap water and stored inside a dark incubator at 37 °C for 7 d.

**Table 1:** The tested RBCs and their compositions according to the manufacturer

Tested RBCs	Composition
Tetric Power Fill (high-viscous bulk-fill)	Bis GMA, UDMA, Bis-EMA, Bis-PMA, DCP, AFCT, Ba-Al-Silicate glass, copolymer, ytterbium trifluoride, mixed oxide
Tetric Prime (high-viscous classic)	Bis GMA, UDMA, Bis-EMA, Ba-Al-Silicate glass, copolymer, mixed oxide, ytterbium trifluoride, silicone dioxide

Bis GMA: Bisphenol A-diglycidyl dimethacrylate, UDMA: Urethane dimethacrylate, Bis-EMA: Ethoxylated bisphenol A dimethacrylate, Bis-PMA: Propoxylated Bisphenol A dimethacrylate, DCP: tricyclodecane-dimethanol dimethacrylate, AFCT: Addition fragmentation chain transfer

After the storage time, impressions were taken for each restoration with an A-silicon material (President Light Body, Coltene Whaledent, Altstatten, Switzerland). The impressions were poured out (Epoxyharz L, R&G Faserverbundwerkstoffe, Waldenbuch, Germany), fixed on aluminium holders (Cementit universal, Merz&Benteli, Niederwangen, Switzerland) and the formed replicas were sputter-coated with gold (Sputter SCD 030, Balzers Union, Balzers, Liechtenstein). After sputter coating, each replica was examined under a stereo microscope (x 20; Zeiss Stemi 1000, Oberkochen, Germany). The cemento-enamel junction buccally and lingually from the restoration was accentuated inside the epoxy resin using a fine scalpel. The replicas were quantitatively analysed for marginal integrity using scanning electron microscopy (SEM) at 20 kV and 200 × magnification (Amray 1810/T, Amray, Bedford, MA, USA). The marginal integrity for each restoration was expressed as a percentage of continuous margins in relation to the entire length of assessable margins. After this initial marginal analysis, all restorations underwent a thermo-mechanical loading (TML) inside an electronic masticator (CoCoM 2, ZPZ, Centre of Dental Medicine, University of Zurich, Zurich, Switzerland). The restorations were loaded on their occlusal part using a metal ball (diameter = 3 mm) for 400 000 loading cycles at 49 N [15]. Simultaneously, the bath temperature within the masticator was changed from 5 to 50 °C with 2 min dwelling time for 1000 times.

**Fig. 2:**
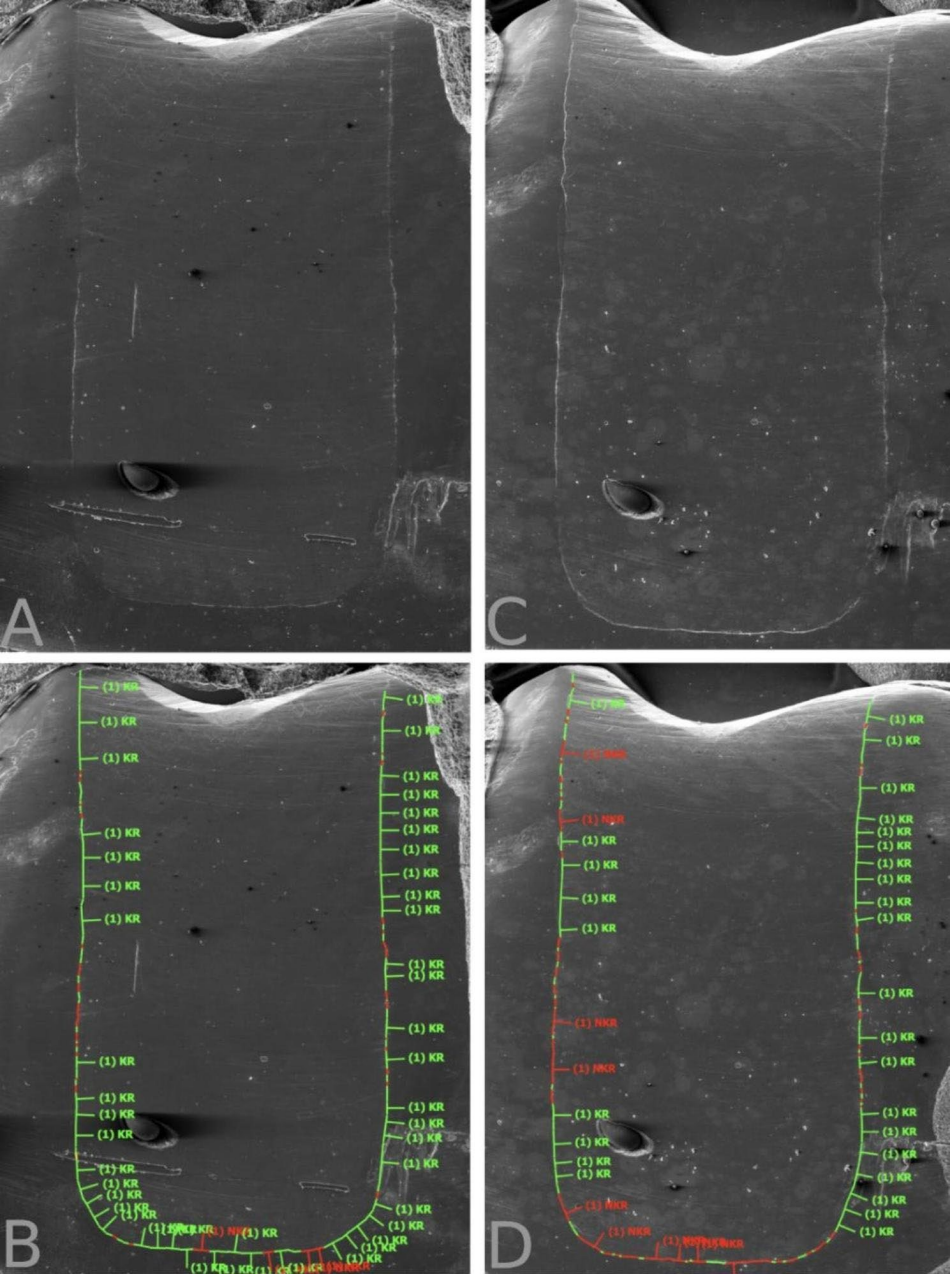
SEM images and the quantitative margin analysis for a restoration’s replica (Power Fill, 10 s). **A**: Status before thermo-mechanical loading (TML). **B**: The marginal analysis before TML. **C**: Status after TML. **D**: The marginal analysis after TML. The green lines indicate continuous margin segments and the red lines non-continuous margin segments

A-silicon impressions were taken again after TML and a final quantitative margin analysis using the same aforementioned protocol was carried out. The margin analysis was conducted by one calibrated and experienced operator (MZ) who was blinded to the groups and had only access to the SEM images. Figure 1 summarises the study protocol and Fig. 2 shows the marginal analysis of a restoration. Under SEM magnification, the cemento-enamel junction on each restoration’s replica was determined. The margins apical to this line (going 1 mm vertically and 3 mm horizontally) were considered in dentine.

### Statistical analysis

The marginal integrity (of the whole restoration, only in enamel and only in dentine) was set as the target variable. This variable was analysed with respect to the restoration type (AFCT-containing bulk-fill RBC “Power Fill” and AFCT-free conventional RBC “Prime”) and the light-curing modes (10 s @ 1200 mW/cm^2^ and 3 s @ 3000 mW/cm^2^). A beta regression model was set to analyse the data. Post-hoc pairwise comparisons between the groups of the same material with different light-curing modes “Power Fill (10 s)” vs. “Power Fill (3 s)” and “Prime (10 s)” vs. “Prime (3 s)” and between the groups of the same light-curing mode with different materials “Power Fill (10 s)” vs. “Prime (10 s)” and “Power Fill (3 s)” vs. “Prime (3 s)” were computed using the emmeans package [16]. The significance level was set at α = 0.05 and data were analysed in R software (The R Foundation for Statistical Computing; Vienna, Austria; www.R-project.org).

## Results

### Marginal integrity of the whole restoration (enamel and dentine)

Before TML, the achieved marginal integrity (% ± standard deviation SD) of each tested RBC (bulk-fill with AFCT “Power Fill” and conventional without AFCT “Prime”) with each tested light-curing mode (regular “10 s” and high-irradiance “3 s”) was as follows: Power Fill (10 s: 92.1 ± 10.2) (3 s: 90.1 ± 5.4), Prime (10 s: 90.1 ± 6.9) (3 s: 90.3 ± 7.3). The difference between “Power Fill (10 s)” vs. “Power Fill (3 s)” and “Prime (10 s)” vs. “Prime (3 s)” and between “Power Fill (10 s)” vs. “Prime (10 s)” and “Power Fill (3 s)” vs. “Prime (3 s)” was not statistically significant (*p* ˃ 0.1).

**Fig. 3:**
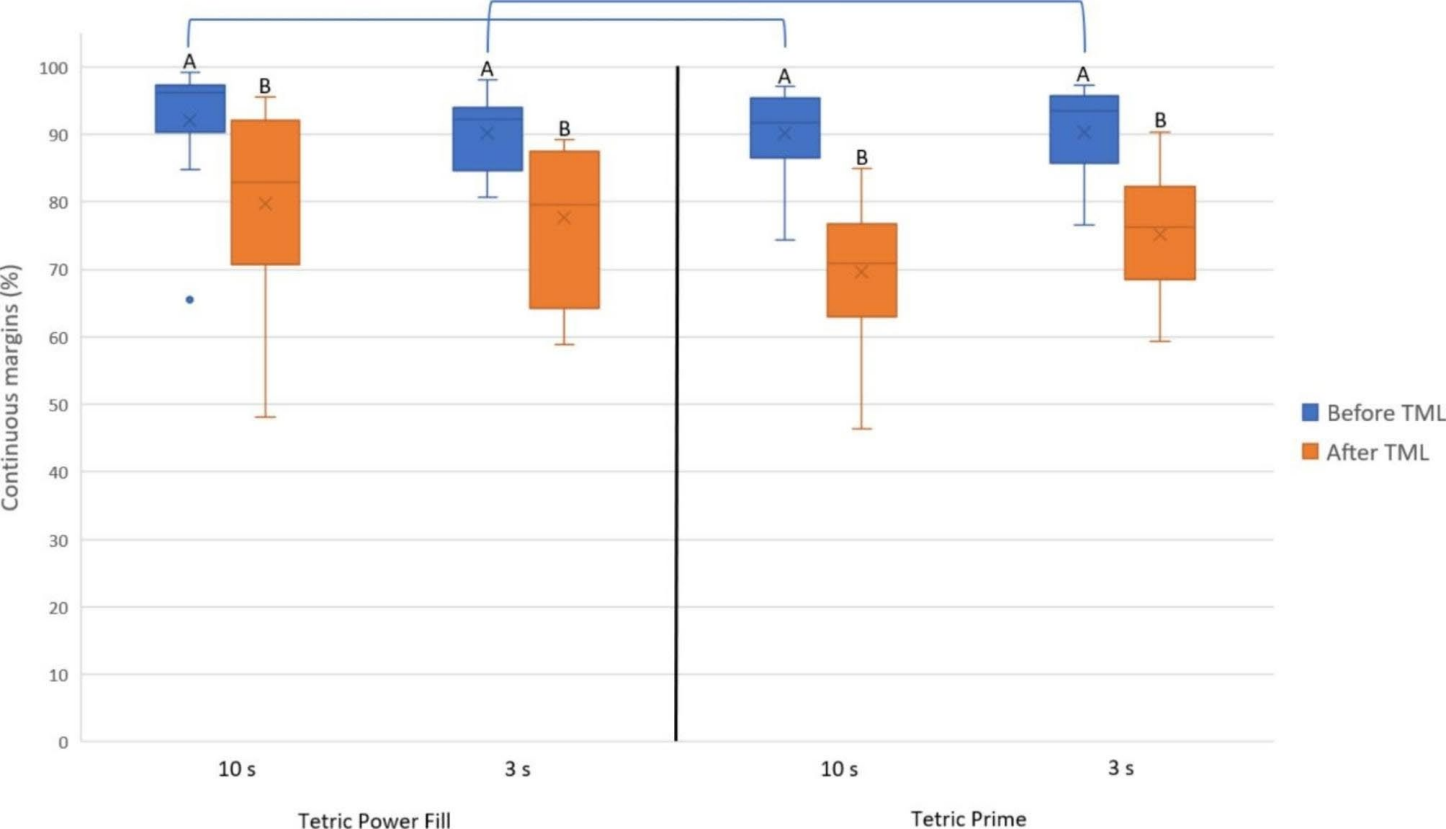
Marginal integrity of the whole restoration in each group before and after TML (means: x marks, medians: horizontal lines, quartiles: boxes, interquartile range: whiskers, outliers: circles). Same capital letters indicate no statistically significant difference between different light-curing modes (high-irradiance: 3 s, regular: 10 s) within the same restorative material. Connecting lines indicate no statistically significant difference between the restorative materials within the same light-curing mode

After TML, the achieved marginal integrity (% ± SD) of each tested RBC with each tested light-curing mode was as follows: Power Fill (10 s: 79.7 ± 15.6) (3 s: 77.6 ± 11.3), Prime (10 s: 69.7 ± 11.1) (3 s: 75.0 ± 9.7). The difference between “Power Fill (10 s)” vs. “Prime (10 s)”, and between “Power Fill (3 s)” vs. “Prime (3 s)” was statistically significant (*p* ≤ 0.05). The difference between “Power Fill (10 s)” vs. “Power Fill (3 s)”, and between “Prime (10 s)” vs. “Prime (3 s)” was not statistically significant (*p* ˃ 0.5). Figure 3 depicts the achieved marginal integrity of the whole restoration and Table 2 represents the regression table of marginal integrity of the whole restoration after TML.

**Table 2:** The regression data analysing the whole restoration after TML

	Coefficient	Standard Error	t value	p value
(Intercept)	1.2958	0.1707	7.59	< 0.001
Light-curing time	-0.0377	0.1851	-0.20	0.84
Material	-0.3803	0.1859	-2.05	0.04

### Marginal integrity of the restoration only within enamel

Before TML, the achieved marginal integrity (% ± SD) of each tested RBC with each tested light-curing mode was as follows: Power Fill (10 s: 93.3 ± 10.6) (3 s: 89.5 ± 5.3), Prime (10 s: 89.3 ± 8.3) (3 s: 91.3 ± 6.7). The difference between “Power Fill (10 s)” vs. “Power Fill (3 s)” and “Prime (10 s)” vs. “Prime (3 s)”, and between “Power Fill (10 s)” vs. “Prime (10 s)” and “Power Fill (3 s)” vs. “Prime (3 s)” was not statistically significant (*p* ˃ 0.1).

**Fig. 4:**
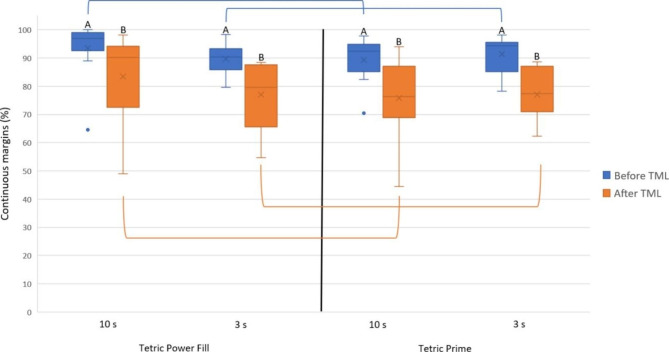
Marginal integrity of the restorations’ parts within enamel before and after TML (means: x marks, medians: horizontal lines, quartiles: boxes, interquartile range: whiskers, outliers: circles). Same capital letters indicate no statistically significant difference between different light-curing modes (high-irradiance: 3 s, regular: 10 s) within the same restorative material. Connecting lines indicate no statistically significant difference between the restorative materials within the same light-curing mode

After TML, the achieved marginal integrity (% ± SD) of each tested RBC with each tested light-curing mode was as follows: Power Fill (10 s: 83.4 ± 16.1) (3 s: 77.0 ± 11.9), Prime (10 s: 75.8 ± 14.1) (3 s: 76.9 ± 9.4). The difference between “Power Fill (10 s)” vs. “Power Fill (3 s)” and “Prime (10 s)” vs. “Prime (3 s)”, and between “Power Fill (10 s)” vs. “Prime (10 s)” and “Power Fill (3 s)” vs. “Prime (3 s)” was not statistically significant (*p* ˃ 0.1). Figure 4 depicts the achieved marginal integrity of the restorations only within enamel.

#### Marginal integrity of the restoration only within dentine

Before TML, the achieved marginal integrity (% ± SD) of each tested RBC with each tested light-curing mode was as follows: Power Fill (10 s: 88.9 ± 14.2) (3 s: 91.3 ± 7.0), Prime (10 s: 92.0 ± 4.7) (3 s: 88.2 ± 13.6). The difference between “Power Fill (10 s)” vs. “Power Fill (3 s)” and “Prime (10 s)” vs. “Prime (3 s)”, and between “Power Fill (10 s)” vs. “Prime (10 s)” and “Power Fill (3 s)” vs. “Prime (3 s)” was not statistically significant (*p* ˃ 0.5).

**Fig. 5:**
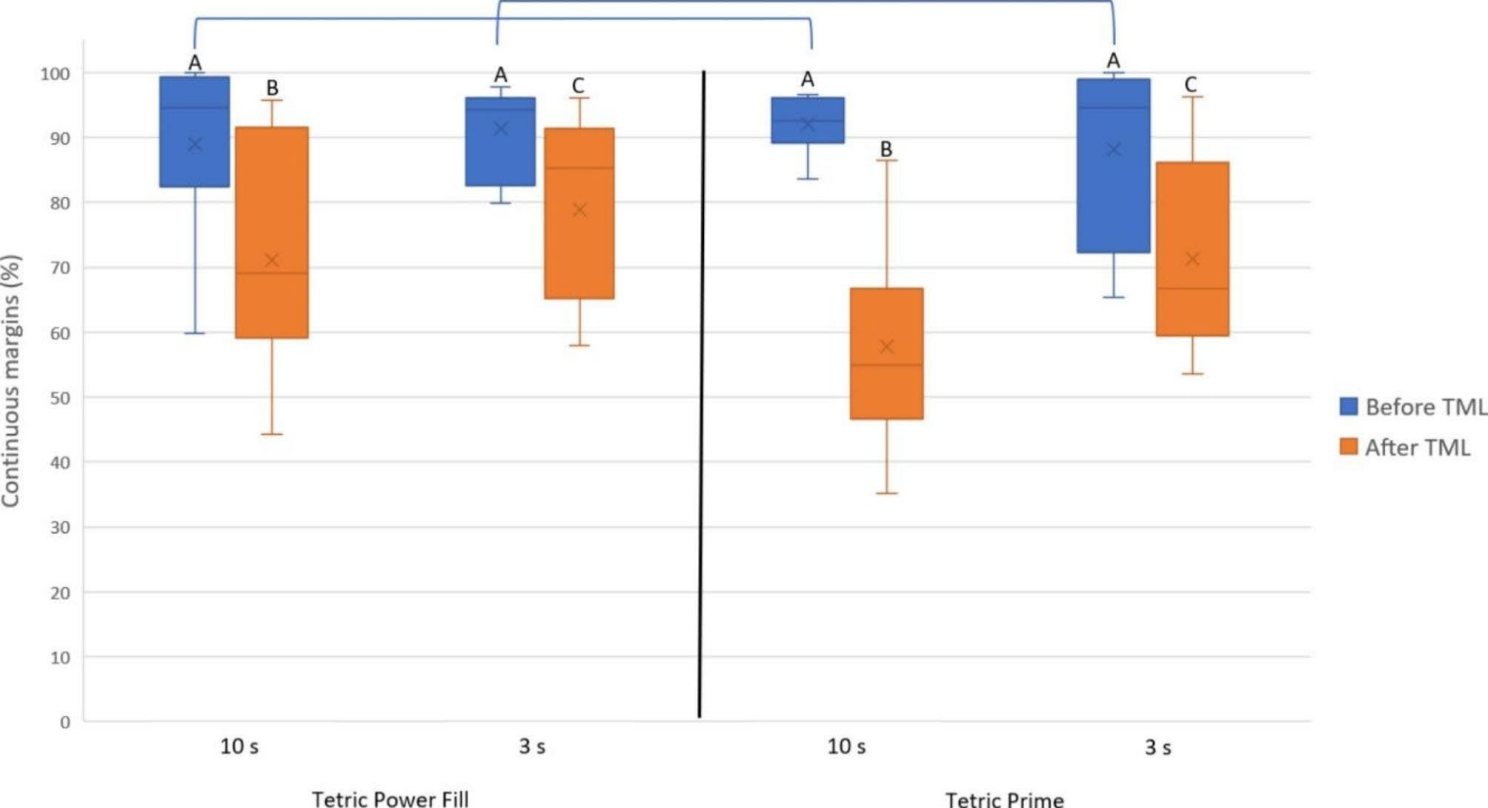
Marginal integrity of the restorations’ parts within dentine before and after TML (means: x marks, medians: horizontal lines, quartiles: boxes, interquartile range: whiskers). Same capital letters indicate no statistically significant difference between different light-curing modes (high-irradiance: 3 s, regular: 10 s) within the same restorative material. Connecting lines indicate no statistically significant difference between the restorative materials within the same light-curing mode

After TML, the achieved marginal integrity (% ± SD) of each tested RBC with each tested light-curing mode was as follows: Power Fill (10 s: 71.0 ± 18.1) (3 s: 78.8 ± 14.0), Prime (10 s: 57.7 ± 15.4) (3 s: 71.2 ± 15.0). The difference between “Power Fill (10 s)” vs. “Prime (10 s)”, and between “Power Fill (3 s)” vs. “Prime (3 s)” was statistically significant (*p* ≤ 0.05). The difference between “Power Fill (10 s)” vs. “Power Fill (3 s)”, and between “Prime (10 s)” vs. “Prime (3 s)” was also statistically significant (*p* ≤ 0.05). Figure 5 depicts the achieved marginal integrity of the restorations only within dentine.

## Discussion

This in-vitro study investigated the effect of a high-irradiance light curing (3 s @ 3000 mW/cm^2^) on the marginal integrity of an AFCT-containing bulk-fill RBC (especially designed for such rapid light curing) and a conventional RBC in standardised deep class-II cavities in primary molars. The marginal integrity in this study was not affected by the different light-curing modes (the first null-hypothesis cannot be rejected), but indeed affected by the tested RBC (the second null-hypothesis must be rejected).

TML strains the adhesive interface between the cavity walls and the restoration and provokes the formation of marginal gaps. As marginal gaps could also form immediately after light-curing due to shrinkage stress of the restorative material, marginal analysis was carried out at two different time points (before and after TML) in the present study. The first attempts to evaluate dental restorative materials with regard to their marginal integrity date back to the 1970s [17]. Some studies reported a correlation between the lack of marginal integrity and marginal discolouration of dental restorations, or even the development of secondary caries underneath them. Other studies, however, stated that this type of correlation is clinically not confirmed, and connected the development of secondary caries to patient related factors rather than the marginal integrity achieved by the restorations [18–20]. The evaluation of marginal integrity in this study was conducted using the indirect impression method. This approach allows assessing margins at different stages throughout the study without damaging the sample (e.g., before and after TML). Nevertheless, it has certain limitations, such as the lack of a standardised impression-taking protocol [21].

The here used loading force and frequency (1.7 Hz, 49 N) is reported to be most frequently used to simulate the mechanical degradation of resin composite restorations in vitro [15]. However, these values are mostly reported for restorations in premolars and permanent molars. Studies investigating composite mechanical degradation in primary teeth are scarce and it is therefore not clear whether other loading forces than those for permanent molars should be recommended. A universal adhesive was used in self-etch mode in this study (without a prior etch-and-rinse step of enamel or dentine). This is a part of simplifying the steps and reducing the time needed for restorative procedures when treating children. A recent study concluded that universal adhesives applied either in selective enamel etch or in self-etch mode result in comparable marginal integrities when restoring deep class-II cavities in primary molars in vitro [22].

Taking the margins of the whole restoration (enamel and dentine) into consideration, employing the high-irradiance light-curing mode resulted in similar marginal integrities as the regular 10-s light-curing mode, regardless of the tested RBC. As for Power Fill, this result could be attributed to the presence of the AFCT reagent, which allows a step-like progression of the polymer chain and enhances the thermal and mechanical properties of RBCs, and to the fact that this RBC was actually developed to be compatible with high-irradiance light curing [13]. Par et al. [23] reported similar results when both light-curing modes were applied to restore class-V cavities in permanent teeth. When light curing a deep restoration for – only – 3 s, concerns can be raised whether a sufficient monomer conversion really takes place, and whether an increased cytotoxicity might result. Two recent studies addressed these issues. Ilie and Watts reported that the degree of conversion of 4-mm-thick layers of Power Fill remained the same when light-cured for 3 or 10 s [9]. The cell toxicity resulting from the same 4-mm-thick layers was also found not to change between both light-curing protocols [24]. Nevertheless, it should be stated that all these tests, including the present study, were conducted in standardised laboratory conditions, where light curing was carried out very attentively. Dentists should be aware of the possible alterations in the clinical situation and prolong the light curing time accordingly. Other material properties of Power Fill (e.g., microhardness, linear shrinkage, shrinkage stress, and micro-tensile bond strength on dentine) were also investigated and found to be similar when employing either high-irradiance or regular light-curing modes [25–28].

The bulk-fill RBC “Power Fill” showed better marginal integrity than the conventional RBC “Prime” regardless of the used light-curing mode. This is in contrast to the results of a recent study, which compared the same RBCs and concluded that both achieve similar marginal integrity in primary molars [5]. This contrast is probably attributed to differences in the cavity design. The class-II cavities in the present study were much deeper and went 1 mm within dentine, whereas the cavities in the other study were – only – 3 mm deep and ended within enamel. Another reason for this superior performance is the fact that the surface of the bulk-fill RBC in this study (placed in 4-mm layer) was constantly closer to the tip of the light-curing unit than the conventional RBC (placed in 2-mm layers), except when light-curing the last layer (the tip was 1 mm away in both materials). Ilie and Watts set the clinical tolerance of the distance between the AFCT-containing RBC and the tip of the light-curing unit at 5 mm [9]. Larger distances than 5 mm, or an angulation of the tip of the light-curing unit, would result in insufficient polymerisation of the AFCT-containing RBC. Due to the small dimensions of primary teeth, it is safe to assume that this distance was never exceeded in the present study, even if the cavity design could be regarded as rather deep for a primary molar. In deeper cavities, where the distance between the light-curing unit and the 4-mm layer of AFCT-containing RBC would be more than 5 mm, an additional 3-s light curing is recommended [9]. To this regard, placing the tip of the light-curing unit exactly where it should be (as near as possible to the restoration and without any angulation) could sometimes be challenging in children due to limited mouth opening and/or lack of sufficient cooperation. Therefore, the additional 3-s light-curing is advisable when the sufficient polymerisation of the restoration is doubtful.

The conventional RBC “Prime” was not especially developed for high-irradiance light curing and does not contain an AFCT reagent. This makes the observed similar marginal integrity between both applied light-curing modes rather interesting. The fact that the radiant exposure in case of the high-irradiance light-curing mode is less than the regular light-curing mode (9.0 vs. 12.0 J/cm^2^) does not actually help explain the observed similar marginal integrity. Nevertheless, Steffen et al. [26] also reported similar micro-tensile bond strength of two RBCs, which were not developed for high-irradiance light curing, when light-cured with regular (10 s @ 1160 mW/cm^2^) or high-irradiance (3 s @ 2850 mW/cm^2^) modes. Similar findings (regarding shrinkage stress and degree of conversion) for AFCT-free RBCs were also reported by Par et al. [25] applying the same abovementioned light-curing modes.

## Conclusions

Based on this in-vitro study and within its limits, it can be concluded that:

- AFCT-containing high-viscous bulk-fill RBC “Power Fill” achieves similar performance with regard to marginal integrity of deep class-II cavities in primary molars when light-cured with high-irradiance (3 s @ 3000 mW/cm^2^) or regular light-curing modes (10 s @ 1200 mW/cm^2^).
- AFCT-containing high-viscous bulk-fill RBC “Power Fill” achieves better performance than the conventional RBC “Prime” with regard to marginal integrity in deep class-II cavities in primary molars regardless of the used light-curing mode.

## Data Availability

The datasets used and/or analysed during the current study are available from the corresponding author on reasonable request.

## Abbreviations

AFCT: Additional fragmentation chain transfer
